# The Clinical Significance of RAS, PIK3CA, and PTEN Mutations in Non-Small Cell Lung Cancer Using Cell-Free DNA

**DOI:** 10.3390/jcm9082642

**Published:** 2020-08-14

**Authors:** Won Jin Chang, Jae Sook Sung, Sung Yong Lee, Eun Joo Kang, Nak-Jung Kwon, Hae Mi Kim, Sang Won Shin, Jung Yoon Choi, Yoon Ji Choi, Ju Won Kim, Kyong Hwa Park, Yeul Hong Kim

**Affiliations:** 1 Division of Oncology/Hematology, Department of Internal Medicine, Korea University Anam Hospital, Korea University College of Medicine, Seoul 02841, Korea; hippo310@hanmail.net (W.J.C.); shinsw@kumc.or.kr (S.W.S.); chspg@hanmail.net (J.Y.C.); yj_choi@korea.ac.kr (Y.J.C.); kohyot35@gmail.com (J.W.K.); khpark@korea.ac.kr (K.H.P.); 2 Cancer Research Institute, Korea University, Seoul 02841, Korea; jsssung@empas.com; 3 Department of Internal Medicine, Korea University Guro Hospital, Seoul 08308, Korea; dragonett@naver.com (S.Y.L.); kkangju11@naver.com (E.J.K.); 4 Macrogen, 254, Beotkkot-ro, Geumcheon-gu, Seoul 08511, Korea; asper76@macrogen.com (N.-J.K.); ham@snu.ac.kr (H.M.K.)

**Keywords:** lung cancer, cell-free DNA, sequencing, mutations

## Abstract

Mutations in the EGFR gene downstream signaling pathways may cause receptor-independent pathway activation, making tumors unresponsive to EGFR inhibitors. However, the clinical significance of RAS, PIK3CA or PTEN mutations in NSCLC is unclear. In this study, patients who were initially diagnosed with NSCLC or experienced recurrence after surgical resection were enrolled, and blood samples was collected. Ultra-deep sequencing analysis of cfDNA using Ion AmpliSeq Cancer Hotspot Panel v2 with Proton platforms was conducted. RAS/PIK3CA/PTEN mutations were frequently detected in cfDNA in stage IV NSCLC (58.1%), and a high proportion of the patients (47.8%) with mutations had bone metastases at diagnosis. The frequency of RAS/PIK3CA/PTEN mutations in patients with activating EGFR mutation was 61.7%. The median PFS for EGFR-TKIs was 15.1 months in patients without RAS/PIK3CA/PTEN mutations, and 19.9 months in patients with mutations (*p* = 0.549). For patients with activating EGFR mutations, the overall survival was longer in patients without RAS/PIK3CA/PTEN mutations (53.8 months vs. 27.4 months). For the multivariate analysis, RAS/PIK3CA/PTEN mutations were independent predictors of poor prognosis in patients with activating EGFR mutations. In conclusion, RAS, PIK3CA and PTEN mutations do not hamper EGFR-TKI treatment outcome; however, they predict a poor OS when activating EGFR mutations coexist.

## 1. Introduction

The identification of targetable genetic driver mutations is a major advancement in non-small cell lung cancer (NSCLC) treatment. Epidermal growth factor receptor (EGFR) mutations are among the most relevant oncogenic alterations. Epidermal growth factor receptor tyrosine kinase inhibitors (EGFR-TKIs) are effective in patients with activating EGFR mutations [1].

The Thr790Met missense mutation (T790M) in the EGFR gene is the major mechanism of acquired drug resistance to EGFR-TKIs in NSCLC [2].

Several studies have reported that gene mutations in the EGFR downstream signal pathways are also significant for the clinical outcome of patients with NSCLC treated with EGFR-TKIs. EGFR activation exerts its effects via the rat sarcoma (RAS)/rapidly accelerated fibrosarcoma (RAF)/mitogen-activated protein kinase (MAPK) and phosphatidylinositol-3-kinase (PI3K)/protein kinase B (AKT) pathways [3].Mutation in the EGFR gene downstream signaling pathways may result in receptor-independent pathway activation, making tumors unresponsive to EGFR inhibitors. KRAS and PI3K are the key regulators of the two aforementioned pathways, respectively [3]. Phosphatase and tensin homolog deleted on chromosome 10 (PTEN), a tumor suppressor gene, dephosphorylates PI-(3, 4, 5)-triphosphate, which mediates AKT activation so as to negatively regulate the PI3K/AKT/mTOR pathway, leading to G1 cell cycle arrest and apoptosis. Therefore, it may also be an important regulator of the EGFR downstream signaling pathways.

KRAS mutations have been demonstrated to predict clinical response to EGFR-TKIs, in addition to serving as poor prognostic factors in patients with NSCLC [4]. PIK3CA mutation is detected in NSCLC with a frequency of 2–7%, and is more frequent in squamous cell lung cancer than in adenocarcinoma [5]. PIK3CA mutation was found both in patients without EGFR-TKI treatment and those resistant to EGFR-TKIs [3]. The occurrence of PTEN mutations in NSCLC is reportedly rare [6]. The clinical significance of these somatic mutations remains unclear because of their low frequencies and the limited information.

The current guidelines and drug-approved companion diagnostics favor a limited single gene assay analysis for EGFR mutation in NSCLC patients, limiting our knowledge of how the most common simultaneous somatic mutations may impact the clinical outcome of EGFR-TKI treatment. The application of next-generation sequencing (NGS) panels that can simultaneously detect multiple gene variants in tumor tissues has increased as a result of the numerous target oncogenes that have been identified. However, the routine use of NGS for tumor genotyping involves some practical challenges. In many cases, tumor tissue samples are not always available for molecular testing at diagnosis or after relapse. Indeed, it has been estimated that about 25% of patients with lung cancer have no tissue available for EGFR testing after completion of histologic assessment [7]. Even when available, they may be of inadequate quality or quantity for mutation testing. In addition, evidence suggests that tumors are highly heterogeneous [8], and the results of a single tumor biopsy may not represent the comprehensive genetic profile of the disease.

The majority of these issues can be overcome by liquid biopsy. Liquid biopsy is a blood test for detecting circulating tumor cells (CTCs) and/or small fragments of cell-free tumor DNA (cfDNA). cfDNA was studied as a potentially valuable surrogate specimen for detecting tumor-specific genetic alterations [9]. As a result, liquid biopsy is entering into clinical practice. In particular, EGFR testing by liquid biopsy for patients with NSCLC has been approved in Europe based on the results of the IFUM trial (Iressa Follow-Up Measure; First-line gefitinib in Caucasian EGFR mutation-positive NSCLC patients: a phase- IV, open-label, single-arm study), which showed an acceptable sensitivity and specificity for this approach [7,10].

In this study, we tested cfDNA with an NGS-based panel using Ion AmpliSeq Cancer Hotspot Panel v2 (ICP; Ion Torrent) with Proton platforms to detect somatic mutations from 126 patients with NSCLC. As described above, mutations in the EGFR gene downstream signaling pathways (RAS/PIK3CA/PTEN) have recently been shown to be associated with the clinical outcome of EGFR-TKI treatment in patients with NSCLC; therefore, we evaluated and focused on these mutations. We correlated the co-mutation profile with the factors of clinical characteristics, response to EGFR-TKI treatment, disease-free survival and overall survival.

## 2. Material and Methods

### 2.1. Patients

All the patients and samples included in this study have been reported in our previous data [11]. Briefly, patients who were initially diagnosed with NSCLC or who experienced recurrence after surgical resection at the Korea University Anam Hospital and Guro Hospital between September 2006 and July 2015 were enrolled in this study. The blood samples and archival formalin-fixed paraffin-embedded pretreatment tumor tissues were collected at the time of initial diagnosis. The blood samples and previous surgically resected specimens were collected at the time of relapse after surgical resection. The clinicopathological parameters and image studies were reviewed. Tumor response was determined in accordance with the Response Evaluation Criteria in Solid Tumors 1.1 (RECIST 1.1) guidelines [12]. This study was approved by the institutional review boards of the Korea University Anam Hospital and Guro Hospital, and informed consent was obtained.

### 2.2. Detection of EGFR Mutations in Tumor Tissues

Tumor tissue genotyping (TTG) for the EGFR gene was performed in the clinical laboratories of the Korea University Anam Hospital and Guro Hospital. The method for detecting EGFR mutations in tumor tissues was described in a previous report [11]. A deletion mutation in exon 19 and point mutation in exon 21 (L858R) were considered activating EGFR mutations.

### 2.3. cfDNA Extraction

As previously described [11], cfDNA was extracted from aliquots (500 µL) of serum with the QIAamp circulating nucleic acid kit (Qiagen, Hilden, Germany) using the QIAvac 24 Plus vacuum manifold (Qiagen, Hilden, Germany), according to the manufacturer’s protocols.

### 2.4. Next-Generation Sequencing (NGS) and Sequencing Data Analysis

The NGS for cfDNA analysis was conducted using Ion AmpliSeq Cancer Hotspot Panel v2 (ICP; Ion Torrent) (Life Technologies, Carlsbad, CA, USA) and the Proton platform, which covers 2800 COSMIC mutations from 50 cancer genes. Further detailed methods are described in our previous study [11]. A variant frequency >0.1% was selected for each sample.

### 2.5. Droplet Digital PCR

To validate the results of ICP, a droplet digital polymerase chain reaction (ddPCR) was carried out with the same cfDNA using the QX200 Droplet Digital PCR (ddPCR) System (BioRad, Milan, Italy). The PrimePCR^TM^ddPCR^TM^ Mutation Assay for humans was used. A commercially available mutation assay kit (BioRad, Milan, Italy) was used to evaluate activating EGFR mutations and KRAS mutations. This kit evaluates EGFR p.E746_ A750del and EGFR WT for p.E746_A750del, EGFR p.T790M and EGFR WT for p.T790M, EGFR p.L858R and EGFR WT for p.L858R, KRAS G12X and KRAS WT for G12X, KRAS G13X and KRAS WT for G13X, and KRAS Q61X and KRAS WT for Q61X, as previously described [11].

### 2.6. Statistical Analysis

All analysis was performed using SPSS 24.0 (SPSS Inc., Chicago, IL, USA). The ICP data from patients with KRAS mutations were validated by ddPCR. On the other hand, the ICP data from patients with NRAS, HRAS, PIK3CA or PTEN mutations were not validated using ddPCR. Survival time was calculated according to the Kaplan–Meier method and compared using log-rank tests. Progression-free survival (PFS) for EGFR-TKI treatment was measured from the first day of treatment with EGFR-TKIs until the date of the last follow-up. Overall survival (OS) was defined as the time from diagnosis to the date of death or the date of the last follow-up. A multivariate analysis was carried out using Cox’ regression analysis to assess the independent prognostic role of each clinical factor. A *p* value < 0.05 was considered statistically significant.

## 3. Results

### 3.1. Detection of Somatic Mutations from cfDNA in 124 Patients with NSCLC

#### 3.1.1. Detection of Activating EGFR Mutations and EGFR T790M Mutation by cfDNA ICP Analysis

EGFR mutations detected by cfDNA ICP analysis and TTG data are presented in Table 1. A total of 14 patients showed activating EGFR mutations only in TTG, while 20 patients showed activating EGFR mutations only in cfDNA. A total of 18 patients had activating EGFR mutations in both analyses. Exon 19 deletion (E19) was found in 23 patients, with a median variant frequency of 1.08% (range, 0.18–44.82%), and the L858R mutation (E21) was identified in 23 patients with a median variant frequency of 0.45% (range, 0.16–28.49%) by ICP analysis. Interestingly, exon 19 deletion and L858R mutation were simultaneously identified in 6 patients by ICP analysis.

EGFR T790M mutation was detected in 29 patients (23.4%) with a median variant frequency of 0.62% (range, 0.19–10.31%) using ICP analysis, but not in the TTG analysis of tumor tissue. In 22 patients, T790M mutation and activating EGFR mutations were simultaneously present, while in 7 patients, no activating EGFR mutations were identified.

#### 3.1.2. Detection of RAS, PIK3CA and PTEN Mutations Using cfDNA ICP Analysis

A total of 33 patients (26.6%) showed RAS mutations using cfDNA ICP analysis. Among them, KRAS mutations were detected in 19 patients (57.6%) with a median variant frequency of 0.22% (range, 0.10–6.72%). NRAS mutations were detected in 13 patients with a median variant frequency of 0.17% (range, 0.14–0.44%), and HRAS mutations were identified in 14 patients with a median variant frequency of 0.21% (range, 0.15–0.81%). Activating EGFR mutations were simultaneously identified in 15 patients (45.5%), and EGFR T790M mutations were present concomitantly in 10 patients (30.3%), as shown by the ICP results.

PIK3CA mutations were detected in 33 patients with a median variant frequency of 0.22% (range, 0.15–3.78%). Among them, 16 patients (48.5%) had simultaneously activating EGFR mutations, and T790M mutations were concomitantly identified in 15 patients (45.5%).

PTEN mutations were identified in 35 patients (28.2%) with a median variant frequency of 0.25% (range, 0.11–0.80%), as determined by ICP analysis. Activating EGFR mutations were simultaneously detected in 17 patients (48.6%), and T790M mutations were present concomitantly in 9 patients (25.7%). PIK3CA mutations coexisted in 14 patients (40.0%) with PTEN mutations.

#### 3.1.3. Validation with ddPCR: EGFR Mutations and KRAS Mutation

In the case of discordant activating EGFR mutations, based on ICP and TTG results, we performed ddPCR to validate the results (Table 1). A total of 14 patients with activating EGFR mutations on TTG results, but not in ICP results, were validated by ddPCR. Among them, activating EGFR mutations were confirmed (>0.01%) in 5 patients. In 4 patients, ddPCR did not detect any EGFR mutations. A total of 20 patients with wild-type EGFR as determined by TTG, but activating EGFR mutations determined by ICP, were also validated using ddPCR. Activating EGFR mutations were detected in 9 patients. Among them, 2 patients had T790M mutations concomitantly, and 4 patients were confirmed as having T790M mutations by ddPCR only. Another 5 patients with wild-type EGFR via TTG, but T790M mutations via ICP, were tested by ddPCR, and ddPCR detected T790M mutations in 4 patients.

Confirmatory ddPCR was also performed in 6 patients with both types of activating EGFR mutations determined by ICP analysis. In total, 2 patients with exon 19 deletions in their TTG results only were positive for exon 19 deletion and L858R in ICP tests, and ddPCR confirmed exon 19 deletions. A total of 1 patient with L858R mutation on their TTG results, but who tested positive for exon 19 deletion and L858R in ICP, was confirmed to have exon 19 deletion by ddPCR.

In the case of mutant KRAS in ICP analysis, we conducted ddPCR to validate the results (Table 2). A total of 19 patients (15.3%) showed mutant KRAS via ICP analysis, and KRAS mutations were confirmed in 13 patients (68.4%) by ddPCR. In 2 patients, confirmatory ddPCR failed, and KRAS mutation was not detected by ddPCR in another 3 patients (G12D, Q61K and G13G in ICP results).

### 3.2. The Clinical Characteristics of the 124 Patients with NSCLC

#### Patients with RAS/PIK3CA/PTEN Mutations

Patients with RAS/PIK3CA/PTEN mutations were analyzed. Out of the total of 124 patients, 72 patients (58.1%) (RAS, *n* = 18; PIK3CA, *n* = 14; PTEN, *n* = 16; RAS and PIK3CA, *n* = 5; RAS and PTEN, *n* = 5; PIK3CA and PTEN, *n* = 8; RAS, PIK3CA and PTEN, *n* = 6) were positive for some of these mutations based on the ICP data. The most frequently mutated sites on KRAS, NRAS, HRAS, PIK3CA and PTEN were G12S, G12D, G12S, H1047R and H61R, respectively. The detailed mutations on each gene were presented in Table S1.

The clinical characteristics according to mutation status are shown in Table 3. The demographics were similar between the patients with mutations and those without mutations, except for stage. Most of the patients with mutations had stage IV NSCLC (*p* = 0.023). A high proportion of the patients with mutations (*n* = 33, 47.8%) had bone metastases at diagnosis, although there was no statistical significance between the two groups. In total, 29 (40.3%) of the patients with mutations had simultaneous activating EGFR mutations. The median OS values for the 72 patients with mutations, and for the 52 patients without mutations, were 13.3 months and 20.5 months, respectively (*p* = 0.666) (Figure S1).

### 3.3. The Clinical Characteristics of Patients with NSCLC with Comprehensive EGFR Activating Mutations

#### Patients with RAS/PIK3CA/PTEN Mutations

**Figure 1:**
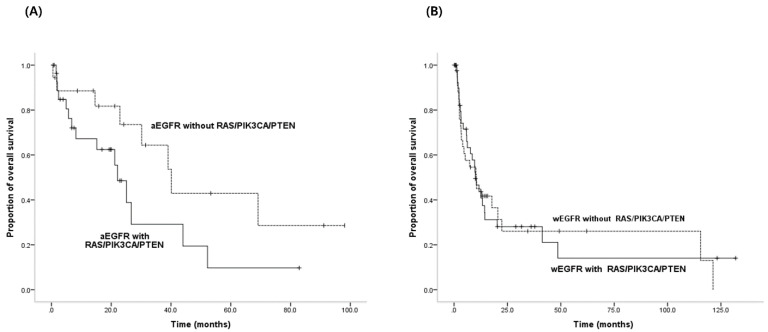
Overall survival in patients with or without RAS/PIK3CA/PTEN mutations according to comprehensive EGFR activating mutational status. (**A**) OS in patients with activating EGFR mutations according to RAS/PIK3CA/PTEN mutations (*p* = 0.080): patients with RAS/PIK3CA/PTEN mutations displayed shorter OS than the patients without mutations. (**B**) OS in patients with wild-type EGFR according to RAS/PIK3CA/PTEN mutations (*p* = 0.932): OS was not different between the two groups.

The frequency of RAS/PIK3CA/PTEN mutations in patients with an activating EGFR mutation was 61.7% (29/47), and in patients with wild-type EGFR it was 55.8% (43/77) (*p* = 0.576). Of the 47 patients with activating EGFR mutations, the patients with RAS/PIK3CA/PTEN mutations showed shorter OS values than the patients without mutations, although there was no statistical significance (median OS (mOS); 22.1 months vs. 40.1 months) (*p* = 0.080) (Figure 1A). Furthermore, of the 77 patients with wild-type EGFR, the OS was not different between the patients with mutations and those without mutations (mOS; 10.0 months vs. 10.4 months) (*p* = 0.932) (Figure 1B).

For the univariate analysis, younger age (≤65 years), female sex, never smoker and activating EGFR mutation were associated with longer survival. For the multivariate analysis, younger age (≤65 years) and activating EGFR mutation were independent good prognostic factors, and RAS/PIK3CA/PTEN mutations were independent predictors of poor prognosis in patients with activating EGFR mutation (Table 4).

### 3.4. EGFR-TKI Treatment Response in Patients Treated with EGFR-TKIs

#### Patients with RAS/PIK3CA/PTEN Mutations

**Figure 2:**
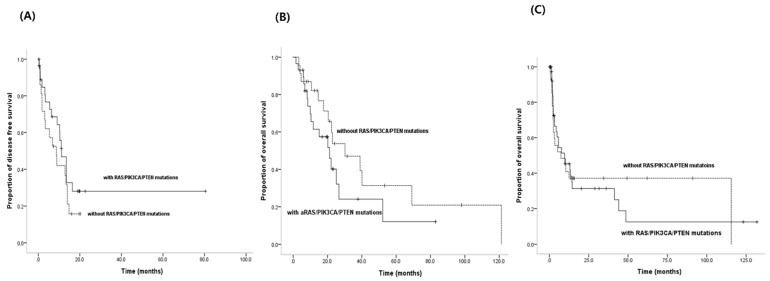
The effect of RAS/PIK3CA/PTEN mutations on clinical outcomes in patients with EGFR TKI treatment. (**A**) Time to progression for EGFR TKIs according to RAS/PIK3CA/PTEN mutations (*p* = 0.246): there was no difference between two groups. (**B**) Overall survival in patients with EGFR TKIs according to RAS/PIK3CA/PTEN mutations (*p* = 0.182): patients with RAS/PIK3CA/PTEN showed shorter OS values. (**C**) Overall survival in patients without EGFR TKIs according to RAS/PIK3CA/PTEN mutations (*p* = 0.836): there was no difference between two groups.

The frequency of having any RAS/PIK3CA/PTEN mutation in patients treated with EGFR-TKIs was 55.8% (29/52), and in patients without EGFR-TKI treatment it was 59.7% (43/72) (*p* = 0.714). Of the 52 patients treated with EGFR-TKIs, the median PFS was 8.7 months in patients without mutations (*n* = 23), and 11.2 months in patients with mutations (*n* = 29) (*p* = 0.246) (Figure 2A). The median OS was 30.2 months in patients without mutations (*n* = 23) and 21.2 months in patients with mutations (*n* = 29) (Figure 2B). Although there was no statistical significance, the patients with mutations showed shorter OS values than those without mutations (*p* = 0.182). Finally, of the 72 patients without EGFR-TKI treatment, the median OS was 7.3 months in patients without mutations (*n* = 29), and 9.6 months in those with mutations (*n* = 43) (*p* = 0.836) (Figure 2C).

### 3.5. PFS for EGFR-TKIs and OS According to both Activating EGFR Mutation Status and Status of RAS/PIK3CA/PTEN Mutations

**Figure 3:**
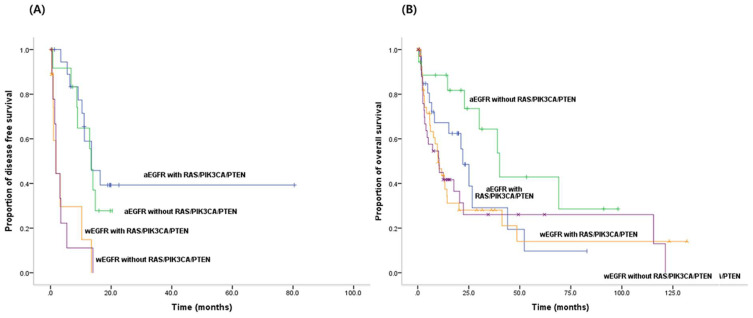
Kaplan–Meier estimates of (**A**) progression free survival (aEGFR vs. wEGFR, *p* < 0.001): patients with activating EGFR mutations had a better PFS for EGFR-TKIs than patients with wild-type EGFR, regardless of the presence or absence of any of RAS/PIK3CA/PTEN mutations, and (**B**) overall survival (aEGFR vs. wEGFR, *p* = 0.022) according to comprehensive EGFR activating mutational status or RAS/PIK3CA/PTEN mutations in NSCLC patients. Morevover, patients with activating EGFR mutations had a better OS than patients with wild-type EGFR, regardless of the presence or absence of any RAS/PIK3CA/PTEN mutations.

We analyzed the patients according to both activating EGFR mutation status and status of RAS/PIK3CA/PTEN mutations. The patients with activating EGFR mutations had a better PFS for EGFR-TKIs (*p* < 0.001), and better OS (*p* = 0.022), than patients with wild-type EGFR, regardless of the presence or absence of any of RAS/PIK3CA/PTEN mutations (Figure 3A,B). The simultaneous presence of RAS/PIK3CA/PTEN mutations did not have a significant effect on PFS in patients with activating EGFR mutations (*p* = 0.549); however, it had a negative effect on OS in patients with activating EGFR mutations, although this effect was not statistically significant (*p* = 0.080).

## 4. Discussion

Molecular profiling for genetic alterations has become a routine practice across the field of clinical oncology [13]. Much of our understanding of genetic profiling is mainly derived from early-stage cancers via TCGA (The Cancer Genome Atlas) data [14]. However, the desire to increase the efficacy of treatment in advanced cancers has led to a recent shift towards “real world” cancer genomics studies focused on real clinical cases [15]. However, to date, information for genetic alteration in advanced cancers has been limited due to the difficulties encountered during the genetic profiling of tumor tissues.

cfDNA was studied as a promising diagnostic sample for detecting tumor-specific genetic alterations in the absence of a tumor tissue [9,16]. Moreover, mutant DNA from tumors at different stages can be obtained from a patient, which may reduce the risk of missing a mutation due to tumor heterogeneity [13]. Several studies investigated the utility of cfDNA in assessing somatic mutations, and reported sensitivities ranging between 58% and 97% [7,17,18]. Furthermore, recent studies reported a relatively high concordance rate between tumor tissue and cfDNA. Arriola et al. reported that the concordance rate for EGFR mutation status between tissue and plasma samples in a cohort of 154 patients with NSCLC was 88.8% [19]. In our previous study, the overall concordance of ICP and TTG analyses was 90% for EGFR exon 19 deletion, and 88% for the L858R mutation [11]. Emerging data have demonstrated the clinical utility of cfDNA from patients as regards the detection of somatic mutations.

In this cohort study, we focused on RAS, PIK3CA or PTEN mutations in cfDNA because these mutations are known to be associated with the clinical outcome of EGFR-TKI treatment and prognosis. Due to the low frequency of RAS, PIK3CA or PTEN mutations in tumor tissue [20], a small number of patients with these mutations had been analyzed in other studies; hence, the clinical significance of these mutations remains unclear. However, in our study, a relatively high proportion of patients with these mutations were analyzed using cfDNA. Our results will be useful for identifying the clinical significance of these mutations, and for making helpful clinical decisions to treat patients with NSCLC.

EGFR-TKIs are the standard treatment for patients with activating EGFR mutations. However, most patients develop acquired resistance, of which 50% is attributed to T790M mutation. Nonetheless, the mechanism of T790M mutation in tumor cells has not yet been clarified. Moreover, many studies have demonstrated that gene mutations in the EGFR downstream signal pathways, such as the RAS/RAF/MAPK and PI3K/AKT/mTOR signaling pathways, are also significantly important in relation to the response to EGFR-TKI treatment in patients with NSCLC. The predictive value of gene mutations in these two pathways downstream of EGFR for EGFR-TKIs is gradually being recognized [3]. KRAS and PI3K are key regulators of these two pathways, and a meta-analysis revealed the independent predictive value of KRAS and PIK3CA mutations in EGFR-TKI treatment [3]. Moreover, these mutations have been described as poor prognostic factors in NSCLC [3,4]. PTEN induces tumor suppression by negatively regulating the PI3K/AKT/mTOR signaling pathways [21]. Furthermore, PTEN is also known to regulate the EGFR pathway. It has been demonstrated in various types of cancer, including lung cancer, that PTEN is mutated to regulate cell survival and progression [22]. In addition, although very limited information of PTEN mutation is available, PTEN mutations are also regarded as being of predictive and prognostic value in patients with NSCLC [22].

Current evidence suggests that KRAS, PIK3CA and PTEN mutations have predictive and prognostic value, although their clinical significance remains unclear; therefore, we analyzed all patients with mutations of RAS, PIK3CA and PTEN using cfDNA. To the best of our knowledge, the clinical outcomes of the EGFR-TKI treatment, and survival, in patients with NSCLC harboring these mutations have not been clarified. Furthermore, the clinical outcome of EGFR-TKI treatment in patients harboring both activating EGFR mutations and RAS, PIK3CA or PTEN mutations has also not been clarified. To the best of our knowledge, our study is the first to clarify these issues.

To validate the results of ICP, ddPCR was carried out with the same cfDNA. However, we only performed the ddPCR of EGFR and KRAS mutations, because commercially available mutation assay kits are limited. Using this approach, we were able to define true and false positives based on concordance between the two tests. This approach could be used to confirm the mutation status of patients who do not have available tissue, and in patients who only have wild-type driver mutations based on TTG [11]. So, more patients will be selected for EGFR-TKIs and other treatments due to comprehensive mutational profiling.

Based on the results obtained from the ICP data, almost half (*n* = 72, 58.1%) of the 124 patients with NSCLC were positive for the RAS, PIK3CA or PTEN mutations. Most of the patients had stage IV NSCLC (88.9%), and a high proportion of patients (*n* = 33, 47.8%) had bone metastases at initial diagnosis. Ryan et al. provided insights into the roles of PI3K in regulating osteoclastic function by demonstrating that PI3K plays an important role in regulating osteoclast morphology and actin cytoskeletal organization [23]. Another study recently reported that PI3K inhibitors inhibited tumor growth in bones, with improved bone structure and bone mineral density in prostate cancer [24]. Furthermore, Cyrille et al. presented the mutational profiling of bone metastases from 44 patients with lung cancer, and showed that KRAS mutations were the most frequent (32% of cases) [25], suggesting that the RAS and PTNE/PIK3CA pathway is related to the metastatic process, especially bone metastasis, rather than primary tumor development.

In our study, RAS/PIK3CA/PTEN mutations did not have a negative effect on PFS in patients with NSCLC treated with EGFR-TKIs. Furthermore, the median OS was not different between patients with these mutations and those without mutations in the 124 patients with NSCLC. Our findings are not consistent with the previously reported data [3,26]. However, patients with RAS/PIK3CA/PTEN mutations had a shorter OS than patients without mutations, when activating EGFR mutations co-existed, although there was no statistical significance (*p* = 0.080). As regards the the multivariate analysis, RAS/PIK3CA/PTEN mutations were found to be poor independent prognostic factors in patients with activating EGFR mutations, consistent with the findings of previous studies, which included only patients with activating EGFR mutations [20].

There are several limitations to this study. First, we only analyzed data from the results of cfDNA. Thus, comparing the results from paired tumor tissues and cfDNA would be more helpful for finding more relevant results. However, as already mentioned, because RAS, PIK3CA and PTEN mutations are more associated with the metastatic process than primary tumor development, there may be the possibility of not detecting these mutations in primary tumor tissues. Second, the results of the NRAS, HRAS, PIK3CA and PTEN mutations obtained from the analysis of cfDNA using ICP could not be validated with the ddPCR method. Furthermore, this study was conducted in only a single center, and a limited number of patients was analyzed. In future, a multicenter study including a large number of patients will be needed.

In conclusion, we demonstrated that ultra-deep sequencing using ICP with a Proton system is a very sensitive method for identifying somatic variants in the cfDNA of patients with NSCLC. Our study suggested that RAS/PIK3CA/PTEN mutations do not have a negative effect on EGFR-TKI treatment outcome, and these mutations predict a poor OS when activating EGFR mutations coexist. Considering the limited clinical information about these mutations in patients with NSCLC, our research is encouraging and valuable, as it provides useful information for understanding these mutations. However, further efforts are needed to improve the clinical outcomes of patients with activating EGFR mutations in whom RAS, PIK3CA or PTEN mutations coexist. 

## Figures and Tables

**Table 1:** Validation of ddPCR in patients with discordant EGFR mutation status between TTG and ICP, and in those with EGFR mutations in ICP analysis, not tested in tumor tissue.

Patient No.	Status of EGFR Mutations		
	TTG	ICP (%)	ddPCR (%)
1	E19	W	0.04
2	E19	W	0.02
3	E19	W	0.00
4	E19	W	0.00
5	E19	W	0.12
6	E19	W	0.00
7	E19	W	0.00
8	E19	W	0.08
9	E19	W	0.00
10	E19	W	39.00
11	E19	W	Failed
12	E21	W	0.01
13	E21	W	0.00
14	E21	W	0.00
15	W	E19 (0.32)	0.00
16	W	E19 (0.70)	0.27
17	W	E19 (7.40)	8.00
18	W	E19 (0.69)	5.60
19	W	E19 (0.67)	0.00
20	W	E19 (0.73)	0.00
21	W	E19 (1.04)	NA
22	W	E19 (0.25), E21 (0.23)	0.03, 0
23	W	E19 (3.34), E21 (0.20)	0.80, 0.12
24	W	E21 (0.32)	0.45
25	W	E21 (0.19)	0.00
26	W	E21 (2.10)	9.20
27	W	E21 (0.68), E20 (0.87)	0.03, 0.05
28	W	E21 (0.45), E20 (0.68)	0.05, 0.06
29	W	E21 (0.65), E20 (2.06)	0, 0.08
30	W	E21 (1.08), E20 (1.24)	NA, 0.29
31	W	E21 (0.58), E20 (0.61)	0.01, 0.03
32	W	E21 (0.25), E20 (0.41)	0, 0.15
33	W	E21 (0.21)	0.01
34	W	E21 (0.63), E20 (0.23)	0, 0
35	W	E20 (0.30)	0.00
36	W	E20 (0.37)	0.11
37	W	E20 (0.50)	0.07
38	W	E20 (0.24)	0.02
39	W	E20 (0.30)	0.03
40	N	E19 (3.11)	4.00
41	N	E19 (1.64)	0.01
42	N	E21 (2.65), E20 (2.41)	0, 0
43	N	E21 (0.36), E20 (0.62)	0, 0.03
44	N	E21 (0.31), E20 (0.57)	0, 0.01
45	N	E21 (1.36), E20 (1.28)	0, 0.20
46	N	E21 (0.25), E20 (0.23)	0, 0.05
47	N	E20 (2.90)	0.03
48	N	E20 (0.22)	0.09
49	N	E21 (0.16), E20 (10.31)	0, 0.53
50	E19	E19 (0.18), E21 (0.16), E20 (0.42)	0.80, 0, 0.23
51	E19	E19 (7.36), E20 (0.24)	17.2, NA
52	E19	E19 (44.82), E21 (3.05), E20 (1.5)	58.00, NA, 0
53	E19	E19 (2.72), E20 (0.97)	4.48, 1.27
54	E21	E21 (0.27), E20 (1.11)	0.07, 0
55	E21	E21 (0.21), E20 (9.56)	0.06, 0.06
56	E21	E21 (0.52), E20 (3.19)	0, 0.04
57	E21	E21 (2.34)	1.69
58	E21	E19 (3.17), E21 (2.32)	0.50, NA
59	E21	E19 (1.08), E20 (1.56)	NA, 0
60	E21	E19 (0.39), E20 (0.19)	0, 0
61	E21	E19 (0.54), E21 (0.21)	0, NA

ddPCR: droplet digital polymerase chain reaction; ICP: Ion AmpliSeq Cancer Hotspot Panel v2; TTG: Tumor tissue genotyping; W: wild type; E19: Exon 19 deletion; E21: Exon 21 mutation (L858R); E20: Exon 20 mutation (T790M); N: Not tested; NA: Not available.

**Table 2:** Validation of ddPCR in patients with mutated KRAS.

Patient No.	Status of KRAS Mutation	
	ICP (%)	ddPCR (%)
1	G13G (0.15)	0.03
2	G12D (1.90)	0.01
3	Q61H (6.72)	28.90
4	G12S (0.46), G13D (0.19), G13G (0.24)	0.05, 0.02, 0
5	Q61K (0.14)	0.00
6	G12S (0.48), G13G (0.19)	NA, NA
7	G12S (0.50), G12C (1.51), G12D (0.15), G13D (0.17), G13G (0.25), Q61H (0.50)	1.07, 1.30, 0.05, 0.01, 0.02, 0.01
8	G12S (0.20)	0.05
9	G12C (0.72)	4.02
10	G13G (0.15)	0.06
11	G12S (0.16)	0.02
12	G13D (0.18)	0.03
13	G12V (0.69)	1.30
14	G12C (0.36)	1.30
15	G13G (0.20)	0.00
16	G12C (0.19)	0.37
17	G12S (0.28)	Failed
18	G13G (0.20)	Failed
19	A59T (0.10)	NA

NA: Not available. A; alanine, C; cysteine, D; aspartate, G; glycine, H; histidine, K; lysine, Q; glutamine, S; serine, T; threonine, V; valine.

**Table 3:** Patients characteristic according to RAS/PIK3CA/PTEN mutations.

	RAS/PIK3CA/PTEN (+) (*n* = 72, %)	RAS/PIK3CA/PTEN (−) (*n* = 52, %)	*p*
Age (years), median	65 (27–87)	64 (42–84)	0.728
Gender			
Male	44 (63.8)	38 (73.1)	0.249
Female	25 (36.2)	14 (26.9)	
Smoking status			
Never smoker	34 (49.3)	28 (53.8)	0.671
Ex-smoker	18 (26.1)	11 (21.2)	
Current smoker	17 (24.6)	13 (25.0)	
Histology			
Adenocarcinoma	52 (75.4)	34 (65.4)	0.121
Squamous carcinoma	7 (10.1)	29 (34.6)	
Others	10 (14.5)		
Stage at sample acquisition			
II	0	1 (1.9)	0.023
III	6 (8.3)	4 (7.7)	
IV	64 (88.9)	38 (73.1)	
Relapsed	2 (2.8)	9 (17.3)	
No. of metastatic organ			
≤2	36 (50)	31 (59.6)	0.362
>2	36 (50)	21 (40.4)	
Bone metastasis			
Present	33 (47.8)	20 (38.5)	0.273
Absent	36 (52.2)	32 (61.5)	
Brain metastasis			
Present	20 (29)	16 (30.8)	0.841
Absent	49 (71)	36 (69.2)	
Activating EGFR mutation			
Present	29 (40.3)	18 (34.6)	0.576
Absent	43 (59.7)	34 (65.4)	

**Table 4:** Overall survival by clinical characteristics; RAS/PIK3CA/PTEN mutations are independent predictors of poor prognosis in patients with activating EGFR mutation.

		Number	Median OS (Months)	*p* (Univariate)	*p* (Multivariate)
Age, years	≤65	67	20.5	0.016	0.003
	>65	57	10.4		
Sex	Male	83	11.7	0.012	0.235
	Female	41	40.1		
Smoking	Yes	59	10.2	0.013	0.235
	No	65	22.9		
EGFR mutations	Wild-type	77	10.4	0.001	<0.001
	Activated	47	30.2		
RAS/PIK3CA/PTEN mutations in activating EGFR mutations	Yes	29	22.1	0.098	0.019
	No	18	40.1		

## Supplementary Materials

The following are available online at https://www.mdpi.com/2077-0383/9/8/2642/s1, Table S1: mutations on RAS, PIK3CA and PTEN gene: out of the total of 124 patients, 72 patients (58.1%) were positive for RAS/PIK3CA/PTEN mutations based on the ICP data. Detailed mutations on each gene were presented. Figure S1: Summary of somatic variations (RAS, PIK3CA and PTEN) in 124 non-small cell lung cancer (NSCLC) patients: most of the patients with RAS/PIK3CA/PTEN mutations had stage IV NSCLC and a high proportion of the patients with mutations had bone and brain metastases at diagnosis. In total, 40.3% of the patients with RAS/PIK3CA/PTEN mutations had simultaneous activating EGFR mutations.
