# Supplementary material for: The Clinical Significance of RAS, PIK3CA, and PTEN Mutations in Non-Small Cell Lung Cancer Using Cell-Free DNA
Source: J Clin Med. 2020 Aug 14;9(8):2642. doi: 10.3390/jcm9082642 (PMC7465200; doi:10.3390/jcm9082642)
Supplement: Supplementary file 1 [file jcm-09-02642-s001.pdf]

Table S1. Mutations on RAS, PIK3CA and PTEN gene: out of the total of 124 patients, 72 patients (58.1%) were positive for RAS/PIK3CA/PTEN mutations based on the ICP data. Detailed mutations on each gene were presented.

Gene	Point mutations	N	Gene	Point mutations	N
**KRAS (n=24)**	G12S	6	**PIK3CA**	H1047R	24
	G12C	4		C420R	14
	G12D	2		E542K	3
	G12V	1		E545K	2
	G13G	3		H1047Y	2
	G13D	3		M1043I	1
	Q13H	1	**PTEN**	H61R	22
	Q61H	1		Y68H	5
	Q61K	1		Q245X	5
	T58I	1		R173C	4
	A59T	1		P246L	4
**NRAS (n=16)**	G12D	10		Q261X	4
	G13D	5		L112P	3
	G60E	1		Q214X	3
**HRAS (n=22)**	G12S	13		H123Y	2
	G12D	4		R130Q	2
	G13D	3		Q17X	2
	G13C	2		Q171X	2
				R335X	2
				G129R	1
				Y16fs	1
				Q298X	1

A; alanine, C; cysteine, D; aspartate, E; glutamate, Fs; frame shift, G; glycine, H; histidine, I; isoleucine, K; lysine, L; leucine, M; methionine, P; proline, Q; glutamine, R; arginine, S; serine, T; threonine, V; valine, X; any amino acid, Y; tyrosine

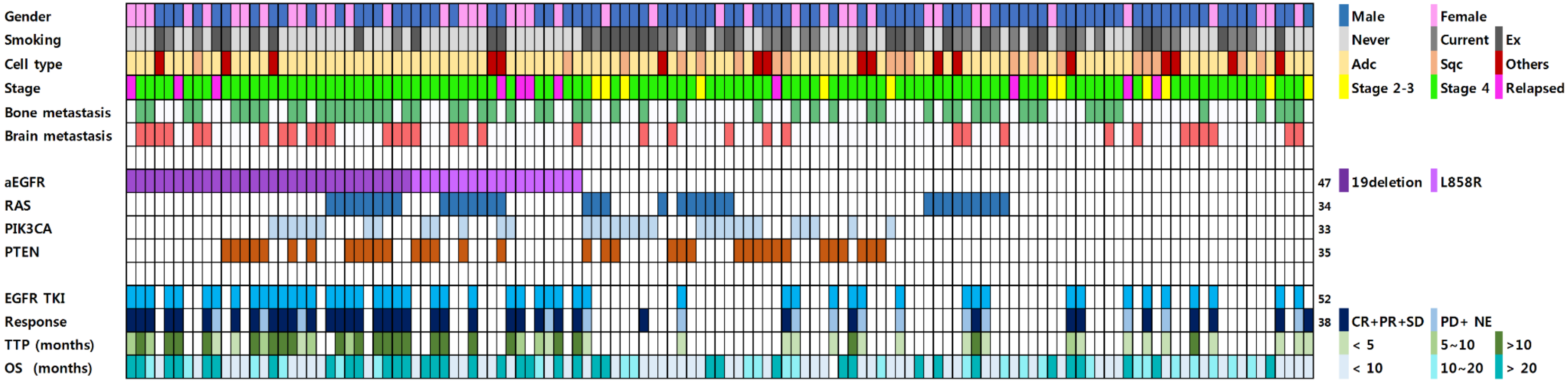

Figure S1. Summary of somatic variations (RAS, PIK3CA and PTEN) in 124 non-small cell lung cancer (NSCLC) patients: most of the patients with RAS/PIK3CA/PTEN mutations had stage IV NSCLC and a high proportion of the patients with mutations had bone and brain metastases at diagnosis. In total, 40.3% of the patients with RAS/PIK3CA/PTEN mutations had simultaneous activating EGFR mutations.
